# A study to investigate the prevalence of headache disorders and migraine conducted using medical claims data and linked results from online surveys: post-hoc analysis of other headache disorders

**DOI:** 10.1186/s12883-024-03675-3

**Published:** 2024-05-25

**Authors:** Takao Takeshima, Koichi Hirata, Hisaka Igarashi, Fumihiko Sakai, Hiromi Sano, Hiroyuki Kondo, Yoshiyuki Shibasaki, Nobuyuki Koga

**Affiliations:** 1 Tominaga Hospital, 1-4-48 Minatomachi, Naniwa-ku, Osaka City, Osaka Japan; 2 Dokkyo Medical University, 880 Kitakobayashi, Mibu, Shimotsuga, Tochigi Japan; 3 Fujitsu Clinic, Kamikodanaka 4-1-1, Nakahara-ku, Kawasaki City, Kanagawa Japan; 4 Saitama Neuropsychiatric Institute, 6-11-1 Honmachi-higashi, Chuo-ku, Saitama City, Saitama Japan; 5 Medical Affairs, Otsuka Pharmaceutical Co., Ltd, 3-2-27, Otedori, Chuo-ku, Osaka City, Osaka Japan; 6 Medical Affairs, Otsuka Pharmaceutical Co., Ltd, 2-6-14, Konan, Minato-ku, Tokyo City, Japan; 7 Present Address: Compliance Department, Otsuka Pharmaceutical Co., Ltd, Tokyo City, Japan; 8 Medical Affairs, Otsuka Pharmaceutical Co., Ltd, 463-10 Kagasuno, Kawauchi-cho, Tokushima City, Tokushima Japan

**Keywords:** Photophobia, Phonophobia, Predictive value, Survey

## Abstract

**Background:**

Surveys using questionnaires to collect epidemiologic data may be subject to misclassification. Here, we analyzed a headache questionnaire to evaluate which questions led to a classification other than migraine.

**Methods:**

Anonymized surveys coupled with medical claims data from individuals 19–74 years old were obtained from DeSC Healthcare Inc. to examine proportions of patients with primary headache disorders (i.e.; migraine, tension-type headache, cluster headache, and “other headache disorders”). Six criteria that determined migraine were used to explore how people with other headache disorders responded to these questions.

**Results:**

Among the 21480 respondents, 7331 (34.0%) reported having headaches. 691 (3.2%) respondents reported migraine, 1441 (6.7%) had tension-type headache, 21 (0.1%) had cluster headache, and 5208 (24.2%) reported other headache disorders. Responses of participants with other headache disorders were analyzed, and the top 3 criteria combined with “Symptoms associated with headache” were “Site of pain” (7.3%), “Headache changes in severity during daily activities” (6.4%), and the 3 criteria combined (8.8%). The symptoms associated with headache were “Stiff shoulders” (13.6%), “Stiff neck” (9.4%), or “Nausea or vomiting” (8.7%), Photophobia” (3.3%) and “Phonophobia” (2.5%).

**Conclusions:**

Prevalence of migraine as diagnosed by questionnaire was much lower than expected while the prevalence of “other headache” was higher than expected. We believe the reason for this observation was due to misclassification, and resulted from the failure of the questionnaire to identify some features of migraine that would have been revealed by clinical history taking. Questionnaires should, therefore, be carefully designed, and doctors should be educated, on how to ask questions and record information when conducting semi-structured interviews with patients, to obtain more precise information about their symptoms, including photophobia and phonophobia.

**Supplementary Information:**

The online version contains supplementary material available at 10.1186/s12883-024-03675-3.

## Highlights

It is difficult to correctly identify “symptoms associated with headache” in a single response survey.

Surveys with questions regarding photophobia and phonophobia should be carefully designed to help headache classification.

It is necessary to educate patients to understand the symptoms of photophobia and phonophobia.

**Supplementary Information:**

The online version contains supplementary material available at 10.1186/s12883-024-03675-3.

## Introduction

Headaches represent the most prevalent of the neurological disorders worldwide, and rank among the leading causes affecting global disability-adjusted life-years [1]. In Japan, headaches are the 5th leading cause of disability in women [2]. The International Classification of Headache Disorders, version 3 (ICHD-3) classifies headaches as primary, where the headache is present in the absence of an underlying pathologic process, disease, or traumatic injury, and secondary, where the headache is due to a causative disorder [3, 4]. The most common primary headaches are migraine, tension-type headache, and cluster headache and other trigeminal autonomic cephalalgias [3, 5]. Other headaches may include primary cough headache, primary exercise headache, primary headache associated with sexual activity, and primary thunderclap headache [3]. The criteria provided by the ICHD-3 has become the accepted standard for diagnosis of headaches [5], and epidemiological studies on migraine in Japan have been conducted based on this classification [6, 7].

Recent epidemiologic studies where primary headache types were based on the ICHD-3 classification provided proportions of the occurrence of these headaches, such as migraine, that were non consistent across subject populations [8, 9]. Our team recently conducted an epidemiological survey linking medical claims and online survey data in order to assess the prevalence of primary headache disorders [10, 11]. The survey classified headache based on the questions presented in the questionnaire, and the responses were used for the evaluation of migraine. In that survey, the prevalence of migraine (including suspected migraine) was 691 (3.2%), that of tension-type headache was 1441 (6.7%), that of cluster headache was 21 (0.1%), and that of other headache disorders 5208 (24.2%) [10]. These findings differ considerably from an earlier survey in Japan that reported a migraine prevalence of 8.4% and the prevalence of “other headaches” as being 8.9% [6], which was greater than expected, and is suspected to include patients with migraine or cluster headache. We, therefore, decided to analyze questionnaire items to assess which questions provided response classifications other than migraine.

## Methods

This is a retrospective observational study using a database comprising medical claims data and survey data. The aim of our study, was to analyze which questionnaire items led to a classification other than migraine. Using a pre-existing survey data, headache disorders were classified into migraine, tension-type, and cluster headaches; and those that were not classified as any of these types, were classified as “other headache disorders”. In this analysis, we focused on migraine and other headache disorders.

Medical claims data and linked survey data obtained by DeSC Healthcare, Inc. (Tokyo, Japan; hereafter, DeSC). DeSC comprised the database of anonymously processed data that was provided by the Society-Managed Employment-Based Health Insurance for the period from 1 December 2017 to 30 November 2020 for subscribers who had agreed to the secondary use of medical data. The study subjects were employees, and their family members, of companies participating in Society-Managed Employment-Based Health Insurance and who were registered users of the health app “kencom®”. Kencom® is a free health monitoring app designed by DeSC that is available, either as a mobile app or via a website, to users in Japan who are members of an affiliated Society-Managed, Employment-Based Health Insurance association [12].

The questionnaire data used for this study were originally collected based on an online questionnaire containing items that were designed to be consistent with the ICHD-3 criteria for migraine and other headache disorders and were administered by DeSC via kencom® from November 1, 2020 to November 30, 2020. This survey consisted of 69 closed-ended questions, with single and multiple possible answers (Supplementary Data 1) [10, 11].

### Eligibility criteria

The study population was derived from approximately 600,000 members of the health insurance association that had contracts with DeSC, and who agreed to the secondary use of medical data. From this population respondents who were aged 19 to 74 years and who were registered in kencom® were provided with online surveys. Only those persons who provided response data for the survey were included in the study. Patients included in this survey were classified as having headache based on the self-administered web questionnaire survey on headache and quality of life (QOL) and based on health insurance association medical claims data.

### Outcome measures

Data were extracted from the database. Survey data included background demographic information, such as gender, age, menstruation status, prefecture of residence, occupation, and annual income. Additionally, the questionnaire items included the presence of headaches and of headache disorders, the frequency, number, and duration of the headaches and their symptoms, headache severity, disruption to daily life, and activities that are interfered with or restricted due to headache. The survey was used to determine if there was a diagnosis of migraine, the status of consultations for migraine and headache, the type of medical institution and department that was consulted, the burden of visiting medical institutions, the presence and nature of symptoms comorbid with migraine/headache, and the status of over-the-counter (OTC) headache medication, including the drug name, frequency of use, and reasons for discontinuation.

The data received from DeSC provided additional background demographic information, the presence or absence of a diagnosis code (i.e.; International Classification of Diseases [ICD]-10 codes) for migraine and/or headache disorder, prescription data for headache medication and for comorbidities, and the presence or absence of diagnosis codes for comorbidities (including hypertension, cardiovascular disease, cerebrovascular disease, gastrointestinal disease, psychiatric or psychosomatic disease, epilepsy, asthma, allergy, autoimmune disease, and other diseases).

### Headache classification

The classification of migraine (Supplementary Data 2) was based on the structured survey responses, including internal diagnostic criteria, as migraine with and without aura, including probable migraine in line with the International Classification of Headache Disorders, 3rd edition (ICHD-3) [5]. Additionally, classification of tension-type headache (Supplementary Data 3) and cluster headache (Supplementary Data 4) were also based on the structured survey responses and classified according to ICHD-3 criteria. Individuals not classified in any of these headache types are included in other headache types.

### Statistical analysis

There were 6 criteria that were used in determining migraine characteristics: Criterion 1: Duration of headache, Criterion 2: Site of pain, Criterion 3: Characteristics of pain, Criterion 4: Change in severity due to daily activities or, State when in pain, Criterion 5: Symptoms associated with headache, Criterion 6: Severity (when not taking medicines). These 6 criteria were tabulated for patients with migraine as well as how individuals who had other headache disorders responded to these questions. The criteria combinations for which there were a large number of cases with headache but were mismatched to migraine (for one, two, and three unsatisfied criteria) were tabulated.

Cases that met the eligibility criteria were included in the analysis population. In this paper we report the results of the analysis population that had headaches. In principle, categorical data are presented as the number of cases and percentage (%). For categorical data, the number of cases and the percentage (%) are shown. Continuous values were summarized by descriptive statistics (N, mean, SD, etc.). The frequency of migraine, other headache disorders were calculated as prevalence rates. The frequency of migraine and other headache disorders were calculated for each disease area distribution, age of onset, and severity. The frequencies of headache diseases, headache medications, and comorbidities, were calculated from receipt information. Statistical significance tests were not conducted. Software used for statistical analysis was SAS Release 9.4 (SAS Institute, Inc., NC, USA).

### Ethics approval and informed consent

The study protocol was approved by the ethics committee of the Research Institute of Healthcare Data Science (approval No.: RI2020012). Additionally, this study was conducted in consideration of the Declaration of Helsinki (revised October 2013) by the World Medical Association and the Ethical Guidelines for Medical Research Involving Human Subjects. This study used only anonymized data provided by DeSC Healthcare, Inc. Moreover, as Otsuka Pharmaceutical Co., Ltd., Clinical Study Support Inc. (CSS), and the medical experts did not possess or receive data correspondence sheets, it was impossible to identify any individual. In addition, DeSC does not have a correspondence table for the data provided to Otsuka Pharmaceutical Co., Ltd, and it is, therefore, impossible to identify individuals from the data provided. Therefore, under the Japanese legal regulations, “Ethical Guidelines for Medical and Health Research Involving Human Subjects”, it was judged unnecessary for the study investigators to obtain new individual level consent for the use of the data in this study.

## Results

### Disposition of respondents

**Fig. 1:**
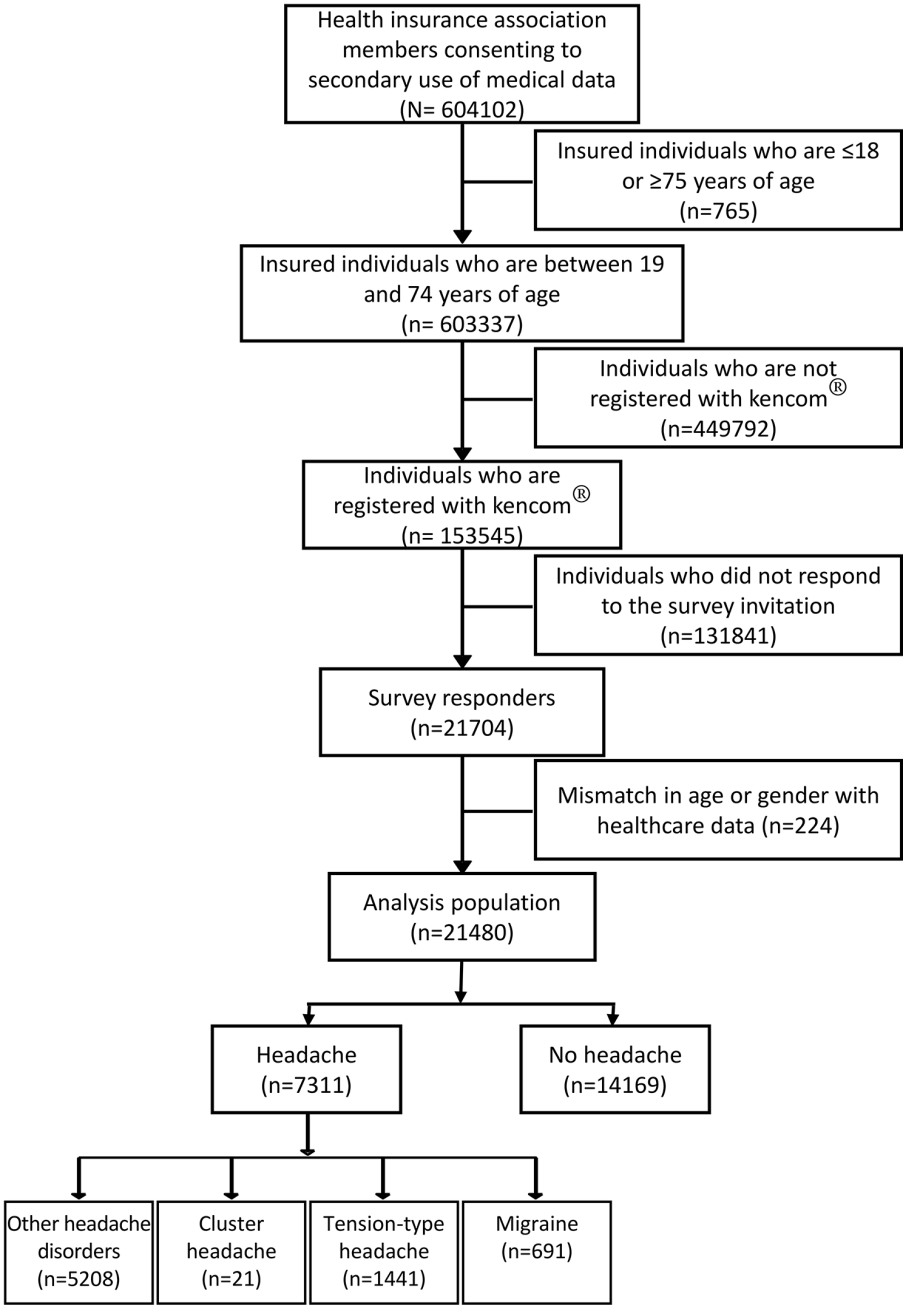
The disposition of participants included in the analyses

There was a total of 604,102 members of the health insurance associations who consented to the secondary use of their medical data (Fig. 1). Of these, 603,337 individuals were between the ages of 19 and 74 years, and of these, 153,545 were registered with kencom®. Of the 21,704 respondents to the survey, 224 were excluded because their age and/or gender did not match the medical claims data, leaving an analyses population of 21,480 respondents. Among the analysis population, 14,169 (66.0%) of the respondents did not have headaches, and 7311 (34.0%) had headache. There were 691 (3.2%) respondents with migraine (including probable migraine), 1441 (6.7%) who had tension-type headache (including those with probable tension-type headache), 21 (0.1%) who had cluster headache, and 5208 (24.2%) who had “other headache disorders”.

### Participant demographic and Disease characteristics

Among those individuals with migraine, 272 (39.4%) were male and 419 (60.6%) were female. Among the 5208 individuals with other headache disorders, 3198 (61.4%) were male and 2010 (38.6%) were female. Most of the participants were between the ages of 30 and 59 years for both groups. There were 667 (96.5%) of participants with migraine who had a headache within the past 45 days and 5065 (97.3%) participants without migraine who reported having had a headache within the past 45 days. Within the comorbidities, gastrointestinal disorders were the most frequently reported comorbidities within the past 6 months in both groups (55.9% for migraine group and 58.1% other headache disorders group). Demographics and disease characteristics are summarized in Table 1.

**Table 1:** Patient demographics and disease characteristics

		Migraine (*N* = 691)	Other headache disorders* (*N* = 5208)
		n (%)	n (%)
Sex^3^	Male	272 (39.4%)	3198 (61.4%)
	Female	419 (60.6%)	2010 (38.6%)
	Menstruation, yes^1^	325 (77.6%)	1439 (71.6%)
Age, years^3^	19–29	60 (8.7%)	284 (5.5%)
	30–39	184 (26.6%)	902 (17.3%)
	40–49	262 (37.9%)	1812 (34.8%)
	50–59	175 (25.3%)	1860 (35.7%)
	60–74	10 (1.4%)	350 (6.7%)
Member status^3^	Insured member	582 (84.2%)	4714 (90.5%)
	Family member	109 (15.8%)	494 (9.5%)
Job category	Professional and technical position	186 (26.9%)	1381 (26.5%)
	Administrative position	47 (6.8%)	722 (13.9%)
	Housewife (or husband)	58 (8.4%)	238 (4.6%)
	Managers	231 (33.4%)	1322 (25.4%)
	Other	169 (24.5%)	1545 (29.7%)
Annual household income^2^ (including tax)	< 1,000,000 JPY (8644 USD)	11 (1.6%)	55 (1.1%)
	≥ 1,000,000 to < 5,000,000 JPY (8644 to 43,220 USD)	136 (19.7%)	1118 (21.5%)
	≥ 5,000,000 to < 10,000,000 JPY (43,220 to 86,440 USD)	360 (52.1%)	2576 (49.5%)
	≥ 10,000,000 JPY (86,440 USD)	119 (17.2%)	1039 (20.0%)
	Don’t know	54 (7.8%)	327 (6.3%)
	No response	11 (1.6%)	93 (1.8%)
Number of days with a headache in the past 3 months	< 45 days	667 (96.5%)	5065 (97.3%)
	≥ 45 days	24 (3.5%)	143 (2.7%)
Number of days with a headache in the past 3 months	Mean (SD)	11.1 (12.3)	8 (12.0)
	Median (min-max)	7 (1–90)	5 (1–92)
Number of days with a headache in the past 30 days	< 15 days	643 (93.1%)	4987 (95.8%)
	≥ 15 days	48 (6.9%)	221 (4.2%)
Number of days with a headache in the past 30 days	Mean (SD)	4.6 (5.0)	3.3 (4.5)
	Median (min-max)	3 (0–30)	2 (0–30)
Receipt code for headache or migraine in the past 6 months^3^	Yes	61 (8.8%)	144 (2.8%)
	No	630 (91.2%)	5064 (97.2%)
Receipt code for headache or migraine in the past 6 months^3^	Migraine	57 (8.2%)	116 (2.2%)
	Tension-type headache	10 (1.4%)	39 (0.7%)
	Cluster headache	0 (0%)	2 (0%)
	Other headache disorders than the above 3 headaches	0 (0%)	1 (0%)
Triptans prescription in the past 6 months		41 (5.9%)	59 (1.1%)
Comorbidity in the past 6 months^3^	Hypertension	51 (7.4%)	633 (12.2%)
	Cardiovascular disorders	23 (3.3%)	287 (5.5%)
	Cerebrovascular disorders	6 (0.9%)	99 (1.9%)
	Epilepsy	6 (0.9%)	39 (0.7%)
	Gastrointestinal disorders	386 (55.9%)	3024 (58.1%)
	Constipation	50 (7.2%)	299 (5.7%)
	Mood disorders	54 (7.8%)	316 (6.1%)
	Anxiety disorders	28 (4.1%)	170 (3.3%)
	Depression	48 (6.9%)	287 (5.5%)
	Asthma	51 (7.4%)	274 (5.3%)

^1^ Denominator was the total number of females

^2^ USD was estimated based on the exchange rate of 1 JPY = 0.008644 USD on 28 January 2022

^3^ Data were derived from the medical claims database. The item “Receipt codes for headache or migraine” was available in the database for individuals who consulted physician and were diagnosed as certain types of headaches

*Headache disorders other than migraine, tension-type headaches or cluster headaches

The responses of the 5208 participants who were classified as other headache disorders (i.e.; other than migraine, tension-type, and cluster headaches) were analyzed to determine which of these criteria had larger percentages of participants who did not match the criteria for migraine based on the questionnaire (Table 2). Among those who had 1 criterion unsuitable, Criterion 5 (Symptoms associated with headache) was mismatched by the largest proportion of participants (52.5%), followed by Criterion 2 (Site of pain: 15.3%), and Criterion 4 (Headache changes in severity during daily activities: 8.7%). The following are the top 3 combinations of criteria in which participants did not match two or more of the criteria in the ICHD-3 questionnaire, along with the percentage of participants for each combination, are:

**Table 2:** Number of participants who did not satisfy the 1, 2, or 3 criteria

	Combinations of unsuitable conditions	Migraine (*N* = 691)	Other headache disorders* (*N* = 5208)
		n (%)	n (%)
1 criterion unsuitable	Duration of headache	23 (3.3%)	0 (0%)
	Site of pain	106 (15.3%)	0 (0%)
	Characteristics of pain	21 (3%)	0 (0%)
	Change in severity due to daily activities or, State when in pain	60 (8.7%)	0 (0%)
	Symptom associated with headache	363 (52.5%)	0 (0%)
	Severity (when not taking medicines)	10 (1.4%)	0 (0%)
2 criteria unsuitable	Duration of headache and Site of pain	0 (0%)	35 (0.7%)
	Duration of headache and Characteristics of pain	0 (0%)	3 (0.1%)
	Duration of headache and Change in severity due to daily activities or, State when in pain	0 (0%)	7 (0.1%)
	Duration of headache and Symptom associated with headache	0 (0%)	152 (2.9%)
	Duration of headache and Severity (when not taking medicines)	0 (0%)	6 (0.1%)
	Site of pain and Characteristics of pain	0 (0%)	32 (0.6%)
	Site of pain and Change in severity due to daily activities or, State when in pain	0 (0%)	92 (1.8%)
	Site of pain and Symptom associated with headache	0 (0%)	381 (7.3%)
	Site of pain and Severity (when not taking medicines)	0 (0%)	17 (0.3%)
	Characteristics of pain and Change in severity due to daily activities or, State when in pain	0 (0%)	14 (0.3%)
	Characteristics of pain and Symptom associated with headache	0 (0%)	76 (1.5%)
	Characteristics of pain and Severity (when not taking medicines)	0 (0%)	1 (0%)
	Change in severity due to daily activities or, State when in pain and Symptom associated with headache	0 (0%)	332 (6.4%)
	Change in severity due to daily activities or, State when in pain and Severity (when not taking medicines)	0 (0%)	12 (0.2%)
	Symptom associated with headache and Severity (when not taking medicines)	0 (0%)	63 (1.2%)
3 criteria unsuitable	Duration of headache, Site of pain and Characteristics of pain	0 (0%)	12 (0.2%)
	Duration of headache, Site of pain and Change in severity due to daily activities or, State when in pain	0 (0%)	27 (0.5%)
	Duration of headache, Site of pain and Symptom associated with headache	0 (0%)	202 (3.9%)
	Duration of headache, Site of pain and Severity (when not taking medicines)	0 (0%)	9 (0.2%)
	Duration of headache, Characteristics of pain and Change in severity due to daily activities or, State when in pain	0 (0%)	5 (0.1%)
	Duration of headache, Characteristics of pain and Symptom associated with headache	0 (0%)	43 (0.8%)
	Duration of headache, Characteristics of pain and Severity (when not taking medicines)	0 (0%)	1 (0%)
	Duration of headache, Change in severity due to daily activities or, State when in pain and Symptom associated with headache	0 (0%)	181 (3.5%)
	Duration of headache, Change in severity due to daily activities or, State when in pain and Severity (when not taking medicines)	0 (0%)	2 (0%)
	Duration of headache, Symptom associated with headache and Severity (when not taking medicines)	0 (0%)	90 (1.7%)
	Site of pain, Characteristics of pain and Change in severity due to daily activities or, State when in pain	0 (0%)	30 (0.6%)
	Site of pain, Characteristics of pain and Symptom associated with headache	0 (0%)	154 (3.0%)
	Site of pain, Characteristics of pain and Severity (when not taking medicines)	0 (0%)	2 (0%)
	Site of pain, Change in severity due to daily activities or, State when in pain and Symptom associated with headache	0 (0%)	459 (8.8%)
	Site of pain, Change in severity due to daily activities or, State when in pain and Severity (when not taking medicines)	0 (0%)	9 (0.2%)
	Site of pain, Symptom associated with headache and Severity (when not taking medicines)	0 (0%)	92 (1.8%)
	Characteristics of pain, Change in severity due to daily activities or, State when in pain and Symptom associated with headache	0 (0%)	102 (2.0%)
	Characteristics of pain, Change in severity due to daily activities or, State when in pain and Severity (when not taking medicines)	0 (0%)	2 (0%)
	Characteristics of pain, Symptom associated with headache and Severity (when not taking medicines)	0 (0%)	28 (0.5%)
	Change in severity due to daily activities or, State when in pain, Symptom associated with headache and Severity (when not taking medicines)	0 (0%)	83 (1.6%)

* Headache disorders other than migraine, tension-type headaches or cluster headaches

- Criterion 5 (Symptoms associated with headache) AND Criterion 2 (Site of pain) AND Criterion 4 (Headache changes in severity during daily activities); 8.8%.
- Criterion 5 (Symptoms associated with headache) AND Criterion 2 (Site of pain); 7.3%.
- Criterion 5 (Symptoms associated with headache) AND Criterion 4 (Headache changes in severity during daily activities); 6.4%.

Among the choices for the symptoms that were associated with Criterion 5 (Symptoms associated with headache: Table 3), “Photophobia” (3.3%) and “Phonophobia” (2.5%) were less common than “Stiff shoulders” (13.6%), “Stiff neck” (9.4%), or “Nausea or vomiting” (8.7%).

**Table 3:** Distribution of responses in each ICHD3 criteria

Question	Response	Migraine (*N* = 691)	Other headache disorders* (*N* = 5208)
		n (%)	n (%)
Criterion 1: Pain duration (single answer)	a) < 4 h	18 (2.6%)	1810 (34.8%)
	b) Half a day	320 (46.3%)	1660 (31.9%)
	c) All day	242 (35.0%)	795 (15.3%)
	d) 2 to 3 days	106 (15.3%)	312 (6.0%)
	e) 4 to 14 days	0 (0%)	40 (0.8%)
	f) ≥ 15 days	0 (0%)	24 (0.5%)
	b) or c) or d) were classified as migraine (ICHD3)	668 (96.7%)	2767 (53.1%)
Criterion 2: Site of pain (multiple answers)	a) Unilateral: ICHD3	585 (84.7%)	1662 (31.9%)
	b) Bilateral	198 (28.7%)	1020 (19.6%)
	c) Frontal	181 (26.2%)	1497 (28.7%)
	d) Occipital	178 (25.8%)	1383 (26.6%)
	e) Periorbital	249 (36.0%)	1152 (22.1%)
	f) Other	17 (2.5%)	249 (4.8%)
Criterion 3: Characteristics (multiple answers)	a) Throbbing or pulsating pain	582 (84.2%)	2411 (46.3%)
	b) Tightening pain	199 (28.8%)	685 (13.2%)
	c) Prickling pain	44 (6.4%)	404 (7.8%)
	d) Tingling pain	27 (3.9%)	246 (4.7%)
	e) Gouged pain behind the eye	158 (22.9%)	365 (7.0%)
	f) Burning pain	3 (0.4%)	18 (0.3%)
	g) Pounding pain	312 (45.2%)	1078 (20.7%)
	h) Cracking pain (like being hit by a hammer)	89 (12.9%)	178 (3.4%)
	i) Heavy-headed	236 (34.2%)	1933 (37.1%)
	j) Other	13 (1.9%)	187 (3.6%)
	At least a) or g) was selected were classified as migraine (ICHD3)	670 (97%)	3023 (58.0%)
Criterion 4 − 1: Change in severity due to daily activities (walking, climbing up-stairs, etc.) or due to physical activity (single answer)	a) Worsens (avoid movement due to pain): ICHD3	360 (52.1%)	546 (10.5%)
	b) No change	138 (20.0%)	1556 (29.9%)
	c) Gets better	28 (4.1%)	581 (11.2%)
	d) Sometimes gets better and sometimes gets worse	73 (10.6%)	429 (8.2%)
	e) I don’t know	92 (13.3%)	2096 (40.2%)
Criterion 4 − 2: State when in pain (single answer)	a) It is more comfortable to stay still: ICHD3	470 (68.0%)	1787 (34.3%)
	b) Staying still does not change the severity of pain	193 (27.9%)	2561 (49.2%)
	c) Pain makes it hard to stay still	22 (3.2%)	79 (1.5%)
	d) I don’t know	6 (0.9%)	781 (15.0%)
For Criterion 5: Symptoms associated with headache	Yes	430 (62.2%)	1175 (22.6%)
	No	261 (37.8%)	4033 (77.4%)
Criterion 5: Symptom associated with headache (multiple answers)	a) Nausea or vomiting	345 (49.9%)	452 (8.7%)
	b) Photophobia	142 (20.5%)	174 (3.3%)
	c) Phonophobia	118 (17.1%)	128 (2.5%)
	d) Osmophobia	91 (13.2%)	92 (1.8%)
	e) Bloodshot eye on the side of headache	11 (1.6%)	21 (0.4%)
	f) Teary eye on the side of headache	27 (3.9%)	18 (0.3%)
	g) Runny nose on the side of headache	17 (2.5%)	28 (0.5%)
	h) Dizziness	101 (14.6%)	217 (4.2%)
	i) Weakness or lethargy	78 (11.3%)	182 (3.5%)
	j) Stiff shoulders	248 (35.9%)	710 (13.6%)
	k) Stiff neck	185 (26.8%)	490 (9.4%)
	l) Numbness in hands and feet	19 (2.7%)	57 (1.1%)
	m) Other	30 (4.3%)	145 (2.8%)
	At least b) and c) are selected were classified as migraine (ICHD3)	26 (3.8%)	24 (0.5%)
	At least a), b) and c) are selected were classified as migraine (ICHD3)	73 (10.6%)	25 (0.5%)
Criterion 6: Severity (when not taking medicines) (single answer)	a) No pain	1 (0.1%)	233 (4.5%)
	b) Little pain	9 (1.3%)	1794 (34.4%)
	c) Moderate pain	316 (45.7%)	2112 (40.6%)
	d) Quite a bit of pain	292 (42.3%)	962 (18.5%)
	e) Extreme pain	73 (10.6%)	107 (2.1%)
	c) or d) or e) is selected was classified as migraine (ICHD3)	681 (98.6%)	3181 (61.1%)
Criterion 4: Criterion 4 − 1 or 4 − 2		631 (91.3%)	1973 (37.9%)

* Headache disorders other than migraine, tension-type headaches or cluster headaches

## Discussion

In the results of this study, 3.2% of respondents had migraine (including probable migraine), and 6.7% of respondents had tension-type headache (including those with probable tension-type headache). These values are considerably lower than the population prevalences which have been reported as 8.4% for migraine [6], and 15.6% for tension-type headache [6]. We believe that the relatively low prevalence of migraine and tension-type headache reported in this study was due to misclassification and resulted from the failure of the questionnaire to identify some features of migraine and tension-type headache that would have been revealed by clinical history taking. Questionnaires should, therefore, be carefully designed, and doctors should be educated on how to ask questions and record information when conducting semi-structured interviews with patients, to obtain precise information about their symptoms, including photophobia and phonophobia.

The present study assessed the criteria that participants selected in describing the properties of their headaches in an effort to determine the type of headache that was experienced by the participant. It is possible that participants in all these other headache disorders do not actually have migraine, but without a physician’s interview, there remains a possibility that the wording or expression of the questions may have led to the classification of other headache disorders. The choice of “unilateral” for Criterion 2 – ‘Site of pain’ – is consistent with migraine, and is one of the diagnostic criteria according to ICHD-3, as one case in point [3]. There are reasons why this criterion (Site of pain) also overlapped with participants with other headache disorders. Participants who have experienced headache episodes over a long period of time may not have been able to choose just one possible item for this criterion (Site of pain), since the sites of the headaches can change when disease duration is long. Moreover, the phenotype of migraine can change over time with the age of the patient and the duration of the disease [13]. For example, one study suggested that the proportion of migraine attacks may decrease with age along with a proportionate increase in attacks of tension-type headaches [13, 14]. In addition, transformation of episodic to chronic migraine can include a pattern of headaches that resemble tension-type headaches [13]. Thus, it may have been difficult for participants with long-duration headache disorder to answer this question in the survey. There is overlap among the different populations experiencing different types of headaches [15]. Patients who have had multiple type of headaches in the past may have difficulty with this criterion (Site of pain) as well. We suggest that when such a questionnaire is administered, or when an interview is conducted by non-specialists, to participants who have had a long duration of headache episodes, it would be helpful to ask about the type and location of headache they have experienced, including if the pain is still continuing at the same location as they have answered in a questionnaire or an interview.

There are possible reasons why the responses to the question Criterion 4 – “Headache changes in severity during daily activities” may be categorized with other headache disorders. A patient may experience more than one type of headache [15], and therefore could not respond accurately. In actual clinical practice, the symptoms associated with headache are taken into account in making the diagnosis of the type of headache. However, this is difficult to do when presenting a uniform questionnaire. Another possible reason is that patients who have experienced multiple headaches may have difficulty into classifying them as one headache because the headache pain could become better or worse by daily activities.

For “nausea“, the response choice of ‘Symptoms associated with headache,’ it will be necessary to ask in detail whether the patient has “upset stomach,” “loss of appetite,” “slight yawn,” etc., because the patient may select “nausea” only when the symptoms are very severe. One possible reason for the lower response rate in participants with migraine to Criterion 5 “photophobia/phonophobia”, under ‘Symptoms associated with headache’ than in past reports [16] may be that respondents had difficulty answering the question and were not confident to select this response. In a study conducted in Japan of outpatients with migraine using semi-structured interviews, 76.5% of patients reported phonophobia and 75.4% reported photophobia [16]. We proposed that, when addressing the symptom of photophobia, the questions should be specific, such as “Do you dim the lights in your room?,” “Do you find it easier when the curtains are closed?,” or “Do you mind the afternoon sun?” Regarding the phonophobia, it will be better to ask questions with specific examples, such as, “When you have a headache, does it bother you if you hear a banging sound, etc.?” or “Do you feel uncomfortable when the TV is on?”

A survey using a comprehensive health literacy questionnaire administered to Japanese participants found that Japanese health literacy is lower than that of Europeans [17]. The authors suggested that this discrepancy may be due in part by certain inefficiencies in the system, and that there is no comprehensive national online platform, making it difficult for Japanese participants to access reliable and understandable health information [17]. Thus, it is possible that Japanese participants may be less familiar with the terms “photophobia” and “phonophobia” than migraine patients may be in other countries. Since the present population is comprised of health insurance association members, their level of knowledge is likely to be higher than that of the general population, but even so, they were not able to successfully answer whether they were photophobia or phonophobia. It is believed that disease awareness was found to be associated with photophobia and phonophobia, among other factors [18]. Improved self-awareness regarding migraine and other headache disorders and of associated symptoms is needed. Because of the diversity of Japanese expressions [19], it is important for non-specialists to distinguish the words appropriately when they conduct a semi-structured interview to diagnose headache. For this purpose, it may be preferable to create a diagnostic tool.

### Limitations

The survey was administered to current employees of companies covered by DeSC contracted health insurance associations or their family members. Members of health insurance associations were large companies with many employees and therefore the survey did not include self-employed workers, civil servants, employees of small and medium-sized companies, or the retired elderly. Employees of companies were expected to have a relatively high socioeconomic status and lived in areas with good medical access and were limited to certain occupations. Thus, there are limitations to generalizing the results of the population in this study to all Japanese adults.

The participants who were targeted to receive the questionnaire were limited to those who were registered users of kencom®. These users tend to be more health-conscious than non-users and more likely to be engaged in healthy living. In general, health-conscious individuals tend to have better health outcomes, thus selection bias may have occurred by underestimating outcomes such as prevalence, resulting in limiting the generalizability.

The survey items of the study were based primarily on self-reported information collected as the questionnaire response, therefore, the ability to recall past medical and prescription histories may have been limited, and the accuracy of the measurement may have been limited. To address this, we obtained highly accurate information on drug prescriptions from the claims data, rather than from the questionnaire. The items related to the physician visit for headache were also evaluated using the claims data as well as the questionnaire. Meanwhile, medical claims data are subject to misclassification because the injury and disease names in the claims data are also insurance disease names for the purpose of medical fee billing.

## Conclusions

When migraine classification was conducted using only a questionnaire without a semi-structured interview, it was clear that some items enabled classification and some items resulted in classification as other than migraine. In the present self-administered online survey, the migraine criterion about symptoms was not satisfied by many of the respondents classified as having other headache disorders, and even by most respondents categorized as having migraine. The results suggest that to capture the subjective and varying experience of headache, particularly symptoms including photophobia and phonophobia, it may be important to carefully devise the survey to help headache classification. In addition, it is necessary to educate patients about headache symptoms so that they understand the symptoms of photophobia and phonophobia. There is also a need for some kind of education for doctors on how to record information when interviewing patients in order to better obtain more precise information about their symptoms.

### Electronic supplementary material

Below is the link to the electronic supplementary material.

Supplementary Material 1

Supplementary Material 2

Supplementary Material 3

Supplementary Material 4

## Data Availability

The data that support the findings of this study are available from DeSC Healthcare, Inc. (Tokyo, Japan) but restrictions apply to the availability of these data, which were used under license for the current study, and so are not publicly available. Data are however available from the authors upon reasonable request and with permission of DeSC Healthcare, Inc.

## Abbreviations

ICHD-3: The International Classification of Headache Disorders, version 3
OTC: Over-the-counter
QOL: Quality of life
ICD: International Classification of Diseases
SD: Standard Deviation
