# Supplementary material for: A study to investigate the prevalence of headache disorders and migraine conducted using medical claims data and linked results from online surveys: post-hoc analysis of other headache disorders
Source: BMC Neurol. 2024 May 25;24:176. doi: 10.1186/s12883-024-03675-3 (PMC11127369; doi:10.1186/s12883-024-03675-3)
Supplement: Supplementary file 1 — Supplementary Material 1 [file 12883_2024_3675_MOESM1_ESM.pdf]

**Supplementary Data 1. English translation of the online survey questions**

**Q1 What is your gender?**

- ○ 1. Male
- ○ 2. Female

**Q2 What is your age?**

- ○ 1. 19 to 29 years
- ○ 2. 30 to 39 years
- ○ 3. 40 to 49 years
- ○ 4. 50 to 59 years
- ○ 5. 60 years or older

**Q3 Females only: Do you have menstrual periods?**

- ○ 1. Yes
- ○ 2. No

**Q4 Where do you live? *If more than one area applies, select the main one.**

- ○ 1. Hokkaido Prefecture
- ○ 2. Aomori Prefecture
- ○ 3. Iwate Prefecture
- ○ 4. Miyagi Prefecture
- ○ 5. Akita Prefecture
- ○ 6. Yamagata Prefecture
- ○ 7. Fukushima Prefecture
- ○ 8. Ibaraki Prefecture
- ○ 9. Tochigi Prefecture
- ○ 10. Gunma Prefecture
- ○ 11. Saitama Prefecture
- ○ 12. Chiba Prefecture
- ○ 13. Tokyo Prefecture
- ○ 14. Kanagawa Prefecture
- ○ 15. Niigata Prefecture
- ○ 16. Toyama Prefecture
- ○ 17. Ishikawa Prefecture
- ○ 18. Fukui Prefecture
- ○ 19. Yamanashi Prefecture
- ○ 20. Nagano Prefecture
- ○ 21. Gifu Prefecture
- ○ 22. Shizuoka Prefecture
- ○ 23. Aichi Prefecture
- ○ 24. Mie Prefecture
- ○ 25. Shiga Prefecture
- ○ 26. Kyoto Prefecture
- ○ 27. Osaka Prefecture
- ○ 28. Hyogo Prefecture
- ○ 29. Nara Prefecture
- ○ 30. Wakayama Prefecture
- ○ 31. Tottori Prefecture
- ○ 32. Shimane Prefecture

- ○ 33. Okayama Prefecture
- ○ 34. Hiroshima Prefecture
- ○ 35. Yamaguchi Prefecture
- ○ 36. Tokushima Prefecture
- ○ 37. Kagawa Prefecture
- ○ 38. Ehime Prefecture
- ○ 39. Kochi Prefecture
- ○ 40. Fukuoka Prefecture
- ○ 41. Saga Prefecture
- ○ 42. Nagasaki Prefecture
- ○ 43. Kumamoto Prefecture
- ○ 44. Oita Prefecture
- ○ 45. Miyazaki Prefecture
- ○ 46. Kagoshima Prefecture
- ○ 47. Okinawa Prefecture
- ○ 48. Other

**Q5 What is your occupation? *If more than one occupation applies, select the main one.**

- ○ 1. Professional or technical position (e.g., doctor, nurse, lawyer, technician, designer, business consultant, interpreter, editor)
- ○ 2. Managerial position (e.g., section chief or higher managerial position, corporate manager or director of a company/organization)
- ○ 3. Administrative position (e.g., accountant, sales administrator, receptionist, secretary, investigator)
- ○ 4. Salesperson
- ○ 5. Sales staff
- ○ 6. Engaged in transportation or communication (e.g., truck or taxi driver, mariner, mail person)
- ○ 7. Engaged in maintenance (e.g., police officer, firefighter, official Self-Defense Forces, security guard)
- ○ 8. Engaged in technical skills or labor (e.g., automobile maintenance, construction, electrical work)
- ○ 9. Engaged in agriculture, forestry, or fisheries (e.g., crop production, livestock raising)
- ○ 10. Service position (requiring a qualification) (e.g., barber/cosmetologist, care worker, cook)
- ○ 11. Service position (not requiring a qualification)
- ○ 12. Student
- ○ 13. Housewife (or husband)
- ○ 14. Part-timer
- ○ 15. No occupation
- ○ 16. Other

**Q6 What is your annual household income (including tax)?**

- ○ 1. 0 yen (no income)
- ○ 2. Less than 1 million yen
- ○ 3. 1 million yen or more and less than 2 million yen
- ○ 4. 2 million yen or more and less than 3 million yen
- ○ 5. 3 million yen or more and less than 4 million yen
- ○ 6. 4 million yen or more and less than 5 million yen
- ○ 7. 5 million yen or more and less than 6 million yen
- ○ 8. 6 million yen or more and less than 7 million yen
- ○ 9. 7 million yen or more and less than 8 million yen
- ○ 10. 8 million yen or more and less than 9 million yen
- ○ 11. 9 million yen or more and less than 10 million yen
- ○ 12. 10 million yen or more and less than 12 million yen

- ○ 13. 12 million yen or more and less than 15 million yen
- ○ 14. 15 million yen or more and less than 20 million yen
- ○ 15. 20 million yen or more
- ○ 16. Don't know

**Q7** **Have you had a headache in the last three months? *Do not include headaches associated with the common cold or a hangover.**

- ○ 1. Yes
- ○ 2. No

**Q8** **How many days did you have a headache in the last three months?**

Approximately [ ] days

**Q9** **Do these headaches occur periodically?**

- ○ 1. Yes
- ○ 2. No

**Q10** **Have any of these headaches ever lasted from seven days to one year?**

- ○ 1. Yes
- ○ 2. No

**Q11** **At about what age did you begin suffering from periodic headaches? Choose the answer that is closest to your own perception.**

- ○ 1. At 5 years of age or younger
- ○ 2. At 6 to 11 years of age
- ○ 3. At 12 to 17 years of age
- ○ 4. At 18 to 19 years of age
- ○ 5. At 20 to 24 years of age
- ○ 6. At 25 to 29 years of age
- ○ 7. At 30 to 39 years of age
- ○ 8. At 40 to 49 years of age
- ○ 9. At 50 to 59 years of age
- ○ 10. At 60 years of age or older
- ○ 11. Don't remember

**Q12** **Are you consulting a doctor for headaches or migraines (hereinafter, "headache") (during the past six months)?**

- ○ 1. Consulting a doctor regularly
- ○ 2. Consulting a doctor though not regularly
- ○ 3. Not consulting a doctor

**Q13** **Have you consulted a doctor for headaches during the past three years? *If you consulted a doctor more than once for testing purposes (including a return to the doctor to hear test results), count these as one consultation.**

- ○ 1. Have consulted a doctor(s) regularly
- ○ 2. Have consulted a doctor(s) several times but not regularly
- ○ 3. Have consulted a doctor only once
- ○ 4. Have not consulted a doctor

**Q14** **Which department are you consulting for the treatment of headaches (during the past six months)? If you are consulting more than one department, select all that apply (multiple choices allowed).**

- ○ 1. General hospital: neurology
- ○ 2. General hospital: neurosurgery

○ 3. General hospital: headache clinic
○ 4. General hospital: internal medicine
○ 5. General hospital: psychosomatic medicine
○ 6. General hospital: ear, nose, and throat
○ 7. General hospital: anesthetics/pain
○ 8. General hospital: obstetrics and gynecology
○ 9. General hospital: pediatrics
○ 10. General hospital: dentistry
○ 11. General hospital: other departments
○ 12. Clinic: neurology
○ 13. Clinic: neurosurgery
○ 14. Clinic: headache clinic
○ 15. Clinic: internal medicine
○ 16. Clinic: psychosomatic medicine
○ 17. Clinic: ear, nose, and throat
○ 18. Clinic: anesthetics/pain
○ 19. Clinic: obstetrics and gynecology
○ 20. Clinic: pediatrics
○ 21. Clinic: dentistry
○ 22. Clinic: other departments

**Q15 Are you receiving treatment for any medical conditions other than headache (during the past six months)?**

○ 1. Yes
○ 2. No

**Q16 What medical conditions are you being treated for, other than headache (during the past six months)? Select all that apply (multiple choices allowed).**

○ 1. Hypertension
○ 2. Heart disease (e.g., mitral valve prolapse, ischemic heart disease, arrhythmia, patent foramen ovale)
○ 3. Cerebrovascular disorder (e.g., ischemic cerebrovascular disorder)
○ 4. Gastrointestinal disorder (e.g., gastrointestinal disorder, constipation)
○ 5. Psychiatric or psychosomatic disorders (e.g., depression, major depression, bipolar disorder, anxiety disorder)
○ 6. Epilepsy
○ 7. Asthma
○ 8. Allergies
○ 9. Autoimmune disorders
○ 10. Other
○ 11. Don't want to answer

**Q17 How many days did you have a headache during the past 30 days? *Do not include headaches associated with the common cold or a hangover.**

Approximately [ ] days

**Q18 Which of the following best describes how long each of these headaches lasts? *If patterns vary, choose the most typical one.**

○ 1. <4 hours
○ 2. Half a day
○ 3. All day
○ 4. 2 to 3 days

- ○ 5. 4 to 14 days
- ○ 6. >=15 days

**Q19** **Where are headaches located? Select all locations that apply (multiple choices allowed).**

- ○ 1. Unilateral
- ○ 2. Bilateral
- ○ 3. Frontal
- ○ 4. Occipital
- ○ 5. Periorbital
- ○ 6. Other

**Q20** **If you chose "Both sides of the head" in the last question, which of the following best describes the frequency of the headache being located on "Both sides of the head?"**

- ○ 1. Every time
- ○ 2. Once every two times
- ○ 3. Less frequent than the above

**Q21** **How would you describe the pain during the headache? Select all that apply (multiple choices allowed).**

- ○ 1. Throbbing or pulsating pain
- ○ 2. Tightening pain
- ○ 3. Prickling pain
- ○ 4. Tingling pain
- ○ 5. Gouged pain behind the eye
- ○ 6. Burning pain
- ○ 7. Pounding pain
- ○ 8. Cracking pain (like being hit by a hammer)
- ○ 9. Heavy-headed
- ○ 10. Other

**Q22** **Does the degree of headache change with activities of daily living (such as walking and going up and down the stairs) and physical activity?**

- ○ 1. Worsens (avoid movement due to pain)
- ○ 2. No change
- ○ 3. Gets better
- ○ 4. Sometimes gets better and sometimes gets worse
- ○ 5. I don't know

**Q23** **Another question about the pain during the headache: Please select the most appropriate one out of the following.**

- ○ 1. It is more comfortable to stay still
- ○ 2. Staying still does not change the severity of pain
- ○ 3. Pain makes it hard to stay still
- ○ 4. I don't know

**Q24** **Do you have any premonitory symptoms (signs) of headache? Select all that apply (multiple choices allowed).**

- ○ 1. Jagged light and/or partial loss of vision
- ○ 2. Numbness of the hand and/or foot on one side
- ○ 3. Partial numbness of the face
- ○ 4. Difficulty speaking
- ○ 5. Weakness on one side

- ○ 6. Other
- ○ 7. No particular signs

**Q25 Do you feel other symptoms along with the headaches?**

- ○ 1. Yes
- ○ 2. No

**Q26 What other symptoms accompany the headaches? Select all that apply (multiple choices allowed).**

- ○ 1. Nausea or vomiting
- ○ 2. Photophobia
- ○ 3. Phonophobia
- ○ 4. Osmophobia
- ○ 5. Bloodshot eye on the side of headache
- ○ 6. Teary eye on the side of headache
- ○ 7. Runny nose on the side of headache
- ○ 8. Dizziness
- ○ 9. Weakness or lethargy
- ○ 10. Stiff shoulders
- ○ 11. Stiff neck
- ○ 12. Numbness in hands and feet
- ○ 13. Other

**Q27 What times of day does a headache usually occur? Select all that apply (multiple choices allowed).**

- ○ 1. Morning/at awakening
- ○ 2. In the morning
- ○ 3. Between noon and evening
- ○ 4. Night
- ○ 5. Other
- ○ 6. No particular patterns

**Q28 When do you usually get a headache? Select all that apply (multiple choices allowed).**

- ○ 1. During work/household chores
- ○ 2. Lack of sleep
- ○ 3. After too much sleep
- ○ 4. While fatigued
- ○ 5. When feeling stressed
- ○ 6. When released from stress
- ○ 7. When feeling nervous
- ○ 8. When released from feeling nervous
- ○ 9. When hungry
- ○ 10. During exercise
- ○ 11. On sunny or rainy days
- ○ 12. In bad weather, such as a typhoon
- ○ 13. When seasons change
- ○ 14. After drinking alcohol
- ○ 15. When smelling perfumes, cigarette, etc.
- ○ 16. When it is related to menstrual periods
- ○ 17. Weekdays
- ○ 18. Holidays (including Saturday and Sunday)
- ○ 19. Before going to bed

- ○ 20. While sleeping
- ○ 21. Other
- ○ 22. No particular conditions under which headaches usually occur

**Q29** **Do you use headache medications? *Medications include both drugs prescribed at medical institutions and nonprescription drugs (over-the-counter drugs).**

- ○ 1. Yes
- ○ 2. No

**Q30** **Which of the following five ratings most closely matches the severity of pain during each headache? *If the severity of pain varies each time, answer for the most typical pattern.**

1. Without medication
   - ○ 1. No pain
   - ○ 2. Little pain
   - ○ 3. Moderate pain
   - ○ 4. Quite a bit of pain
   - ○ 5. Extreme pain

2. With medication (*If the severity of pain varies with medication, answer for the most typical pattern.)
   - ○ 1. No pain
   - ○ 2. Little pain
   - ○ 3. Moderate pain
   - ○ 4. Quite a bit of pain
   - ○ 5. Extreme pain

**Q31** **Which of the following most closely matches the degree of interference with your daily activities by each headache? *If the degree of interference varies with each headache, answer for the most typical pattern.**

1. Without medication
   - ○ 1. No interference with daily activities at all
   - ○ 2. Mild interference with daily activities
   - ○ 3. Moderate interference with daily activities
   - ○ 4. Severe interference with daily activities
   - ○ 5. Extreme interference with daily activities

2. With medication (*If the severity of interference varies with medication, answer for the most typical pattern.)
   - ○ 1. No interference with daily activities at all
   - ○ 2. Mild interference with daily activities
   - ○ 3. Moderate interference with daily activities
   - ○ 4. Severe interference with daily activities
   - ○ 5. Extreme interference with daily activities

**Q32** **Select all nonprescription drugs (over-the-counter drugs) that you are taking for the treatment of headache out of the following (during the past six months). In addition, select all nonprescription drugs that you are not currently taking but have taken previously (multiple choices allowed).**

1. Nonprescription drugs that you are taking for the treatment of headaches (during the past six months)
   - ○ 1. LOXONIN S
   - ○ 2. LOXONIN S PREMIUM
   - ○ 3. BUFFERIN EX
   - ○ 4. Loxoprofen Tablets
   - ○ 5. LUMIFEN

- ○ 6. RINGL IB α200
- ○ 7. EVE QUICK
- ○ 8. BUFFERIN PREMIUM
- ○ 9. Norshin AI
- ○ 10. Wilquest IPa
- ○ 11. TYLENOL A
- ○ 12. SEDES HIGH
- ○ 13. SEDES HIGH G
- ○ 14. Saridon Wi
- ○ 15. SEMIDON GRANULES
- ○ 16. Kerorin
- ○ 17. Hakkiri Ace a
- ○ 18. Traditional Chinese medicines
- ○ 19. Other
- ○ 20. Not taking/have not taken any of these

2. Nonprescription drugs that you have previously taken for the treatment of headaches
   - ○ 1. LOXONIN S
   - ○ 2. LOXONIN S PREMIUM
   - ○ 3. BUFFERIN EX
   - ○ 4. Loxoprofen Tablets
   - ○ 5. LUMIFEN
   - ○ 6. RINGL IB α200
   - ○ 7. EVE QUICK
   - ○ 8. BUFFERIN PREMIUM
   - ○ 9. Norshin AI
   - ○ 10. Wilquest IPa
   - ○ 11. TYLENOL A
   - ○ 12. SEDES HIGH
   - ○ 13. SEDES HIGH G
   - ○ 14. Saridon Wi
   - ○ 15. SEMIDON GRANULES
   - ○ 16. Kerorin
   - ○ 17. Hakkiri Ace a
   - ○ 18. Traditional Chinese medicines
   - ○ 19. Other
   - ○ 20. Not taking/have not taken any of these

**Q33** **What are your reasons for not taking any nonprescription drugs (over-the-counter drugs) (during the past six months)? Select all that apply (multiple choices allowed).**

- ○ 1. Headaches have been relieved
- ○ 2. Medications were not effective
- ○ 3. Could not continue with medications owing to side effects
- ○ 4. Financial burden of medications was significant
- ○ 5. Burden of taking medications every day was significant
- ○ 6. Often missed doses owing to being busy with work, household chores, schoolwork, etc.
- ○ 7. Other

**Q34** **Which of the following troubles do you have due to interference with daily activities by headaches? Select all that apply (multiple choices allowed).**

- ○ 1. Difficulty concentrating on (slowing down) work or study
- ○ 2. Feeling unmotivated for work or study
- ○ 3. Sometimes taking time off work or study
- ○ 4. Difficulty taking public transportation
- ○ 5. Difficulty being at a busy place
- ○ 6. Difficulty/unmotivated for doing household chores
- ○ 7. May want to but have difficulty putting on makeup
- ○ 8. Difficulty going out (e.g., driving children to and from school, going shopping, keeping relationships with neighbors)
- ○ 9. Sometimes canceling plans/appointments
- ○ 10. Difficulty scheduling time for work and private life
- ○ 11. Difficulty having daily conversations with family and friends, etc.
- ○ 12. Difficulty socializing with friends and playing with children
- ○ 13. Difficulty driving a car
- ○ 14. Difficulty getting understanding from those around me
- ○ 15. Other

**Q35** **Which of the following most closely matches the decrease in the frequency of headaches interfering with daily activities that would improve your daily activities? Choose one.**

- ○ 1. Even a slight decrease
- ○ 2. Decrease to about half
- ○ 3. Almost disappearing
- ○ 4. Decrease in the pain of each headache rather than decreased frequency

**Q36** **Do you have any reduced activities, i.e. the activities themselves or the frequency, owing to headache? Select all that apply (multiple choices allowed).**

- ○ 1. Laundry
- ○ 2. Cooking (cooking for yourself/your family)
- ○ 3. Going to grocery shopping
- ○ 4. Housework (excluding grocery shopping, laundry, and cooking)
- ○ 5. Putting on makeup
- ○ 6. Bathing
- ○ 7. Driving children or other family members to and from school/activities
- ○ 8. Socializing with friends and playing with children
- ○ 9. Keeping relationships with neighbors
- ○ 10. Exercising such as playing sports or walking
- ○ 11. Going to crowded places
- ○ 12. Drinking alcohol
- ○ 13. Taking public transportation
- ○ 14. Driving a car
- ○ 15. Operating a computer or smart phone
- ○ 16. Other

**Q37** **Which of the following most closely matches the frequency that you are consulting a doctor for headaches (during the past six months)?**

- ○ 1. Once a week or more
- ○ 2. About once every 2 weeks
- ○ 3. About once a month
- ○ 4. About once every 2 months

- ○ 5. About once every 3 months
- ○ 6. Less frequently than the above

**Q38** **What is the degree of your current time-wise burden due to the frequency of visiting a hospital/clinic (during the past six months)?**

- ○ 1. Do not feel any burden
- ○ 2. Do not feel much burden
- ○ 3. No opinion
- ○ 4. Feel a slight burden
- ○ 5. Feel a considerable burden

**Q39** **Another question: What is the degree of your current financial burden due to the frequency of visiting a hospital/clinic (during the past six months)?**

- ○ 1. Do not feel any burden
- ○ 2. Do not feel much burden
- ○ 3. No opinion
- ○ 4. Feel a slight burden
- ○ 5. Feel a considerable burden

**Q40** **If you were regularly visiting a hospital/clinic during the past three years, which of the following best describes the frequency of visiting a hospital/clinic for headaches? *If you were regularly visiting a hospital/clinic during different time periods, answer the frequency of the most recent regular visiting.**

- ○ 1. Once a week or more
- ○ 2. About once every 2 weeks
- ○ 3. About once a month
- ○ 4. About once every 2 months
- ○ 5. About once every 3 months
- ○ 6. Less frequently than the above

**Q41** **What is the degree of your time-wise burden due to the frequency of visiting a hospital/clinic during the past three years?**

- ○ 1. Do not feel any burden
- ○ 2. Do not feel much burden
- ○ 3. No opinion
- ○ 4. Feel a slight burden
- ○ 5. Feel a considerable burden

**Q42** **Another question: What is the degree of your financial burden due to the frequency of visiting a hospital/clinic during the past three years?**

- ○ 1. Do not feel any burden
- ○ 2. Do not feel much burden
- ○ 3. No opinion
- ○ 4. Feel a slight burden
- ○ 5. Feel a considerable burden

**Q43** **What made you consult a doctor for headaches? Select all that apply (multiple choices allowed).**

- ○ 1. Increased headache frequency
- ○ 2. Unable to tolerate headaches
- ○ 3. OTC analgesics no longer effective
- ○ 4. Worried about other brain diseases
- ○ 5. Recommendation by my family, friend, or another person around me
- ○ 6. Recommendation by a doctor or someone who saw me for another disease

- ○ 7. Got to know someone who was consulting a doctor for headaches
- ○ 8. Learned about a "headache clinic"
- ○ 9. Information on the Internet made me feel like consulting a doctor
- ○ 10. Information from TV, newspapers, magazines, etc. made me feel like consulting a doctor
- ○ 11. Other

**Q44** **If you consulted a doctor only once during the past three years, what are your reasons for not consulting a doctor thereafter? Select all reasons that apply (multiple choices allowed).**

- ○ 1. Symptoms improved after consulting a doctor
- ○ 2. Relieved not to have a brain disease that threatened life
- ○ 3. Want to visit a hospital but have no time
- ○ 4. Visiting a hospital is troublesome
- ○ 5. The doctor who saw me was not kind
- ○ 6. The prescribed medications did not work
- ○ 7. Because of the financial burden
- ○ 8. Other

**Q45** **If you did not consult a doctor for headaches during the past three years, what are the reasons? Select all reasons that apply (multiple choices allowed).**

- ○ 1. Used to having a headache
- ○ 2. Spontaneously resolving after endurance
- ○ 3. OTC drugs can reduce the pain
- ○ 4. Did not think that headaches were a disease that necessitates visiting a hospital
- ○ 5. Want to visit a hospital but have no time
- ○ 6. Want to visit a hospital but have no idea about the hospital/department that I should visit
- ○ 7. Because of the financial burden
- ○ 8. Symptoms of headache occurred only recently
- ○ 9. The pain was not so severe as to require visiting a hospital
- ○ 10. Other

**Q46** **Have you ever been diagnosed with "migraine" at a medical institution that you visited for headaches?**

- ○ 1. Yes
- ○ 2. No

**Q47** **Are you taking any headache medications prescribed at a medical institution (during the past six months)?**

- ○ 1. Taking prescribed headache medication(s)
- ○ 2. Currently not taking any but was previously taking prescribed headache medication(s)
- ○ 3. Have not taken any prescribed headache medications

**Q48** **What is the current status of headache medications prescribed at a medical institution (during the past six months)?**

- ○ 1. Taking such medications regularly and when having headaches
- ○ 2. Taking such medications only when having headaches
- ○ 3. Missing doses of such medications often/rarely taking such medications
- ○ 4. Other

**Q49** **If you are not taking prescription drugs but were previously taking headache medication(s) prescribed at a medical institution, what are the reasons? Select all reasons that apply (multiple choices allowed).**

- ○ 1. Headaches have been relieved

- ○ 2. Medications were not effective
- ○ 3. Could not continue with medications owing to side effects
- ○ 4. Financial burden of medications was significant
- ○ 5. Burden of taking medications every day was significant
- ○ 6. Often missed doses owing to being busy with work, household chores, schoolwork, etc.
- ○ 7. Medications were discontinued for drugs prescribed for other diseases
- ○ 8. Other

**Q50-Q63: Migraine-Specific Quality-Of-Life Questionnaire version 2.1** [1-3]

**Q64-Q69: Work Productivity and Activity Impairment Questionnaire: General Health** [4]

1. GlaxoSmithKline Research and Development Limited (GSK). Migraine-Specific Quality-of-Life Questionnaire (MSQ Version 2.1). 1992. https://eprovide.mapi-trust.org/instruments/migraine-specific-quality-of-life-questionnaire. Accessed 11 Jan 2024.
2. Rendas-Baum R, Bloudek LM, Maglinte GA, Varon SF. The psychometric properties of the Migraine-Specific Quality of Life Questionnaire version 2.1 (MSQ) in chronic migraine patients. Qual Life Res. 2013;22:1123-33.
3. Speck RM, Shalhoub H, Ayer DW, Ford JH, Wyrwich KW, Bush EN. Content validity of the Migraine-Specific Quality of Life Questionnaire version 2.1 electronic patient-reported outcome. J Patient Rep Outcomes. 2019;3:39.
4. Reilly MC, Zbrozek AS, Dukes EM. The validity and reproducibility of a work productivity and activity impairment instrument. Pharmacoeconomics. 1993;4:353-65.
