# Supplementary material for: A study to investigate the prevalence of headache disorders and migraine conducted using medical claims data and linked results from online surveys: post-hoc analysis of other headache disorders
Source: BMC Neurol. 2024 May 25;24:176. doi: 10.1186/s12883-024-03675-3 (PMC11127369; doi:10.1186/s12883-024-03675-3)
Supplement: Supplementary file 2 — Supplementary Material 2 [file 12883_2024_3675_MOESM2_ESM.docx]

## **Supplementary Data 2** Classification of migraine

| **Item^1^** | **Response** | **Patterns of responses** |
| --- | --- | --- |
| Duration (single answer) | a) <4 hours  b) Half a day  c) All day  d) 2 to 3 days  e) 4 to 14 days  f) ≥15 days | At least b), c), or d) is selected |
| Site of pain (multiple answers) | a) Unilateral  b) Bilateral  c) Frontal  d) Occipital  e) Periorbital  f) Other | At least a) is selected |
| Characteristics (multiple answers) | a) Throbbing or pulsating pain  b) Tightening pain  c) Prickling pain  d) Tingling pain  e) Gouged pain behind the eye  f) Burning pain  g) Pounding pain^2^  h) Cracking pain (like being hit by a hammer)  i) Heavy-headed  j) Other | At least a) or g) is selected |
| Change in severity due to daily activities (walking, climbing up-stairs, etc.) or due to physical activity (single answer) | a) Worsens (avoid movement due to pain)  b) No change  c) Gets better  d) Sometimes gets better and sometimes gets worse  e) I don't know | At least a) in “Change in severity due to daily activities” or a) in “State when in pain” is selected |
| State when in pain (single answer) | a) It is more comfortable to stay still  b) Staying still does not change the severity of pain  c) Pain makes it hard to stay still  d) I don't know |  |
| Symptom associated with headache (multiple answers) | a) Nausea or vomiting  b) Photophobia  c) Phonophobia  d) Osmophobia  e) Bloodshot eye on the side of headache  f) Teary eye on the side of headache  g) Runny nose on the side of headache  h) Dizziness  i) Weakness or lethargy  j) Stiff shoulders  k) Stiff neck  l) Numbness in hands and feet  m) Other | a)  b) and c)  a), b), and c)  One of the above combinations is selected |
| Severity (single answer) | a) No pain  b) Little pain  c) Moderate pain  d) Quite a bit of pain  e) Extreme pain | At least one of c), d), or e) is selected |

**Notes:** This study used the classification used in Sakai et al. [1].

^1^ If only one of the six criteria above did not apply, the patient was considered to have a "probable migraine" and was included in the migraine category.

**Reference**

1. Sakai F, Hirata K, Igarashi H, Takeshima T, Nakayama T, Sano H, et al. (submitted) A survey conducted using health claims records and questionnaires to investigate the prevalence of migraine in Japan. [Submitted to the Journal of Headache and Pain].
