# Supplementary material for: A study to investigate the prevalence of headache disorders and migraine conducted using medical claims data and linked results from online surveys: post-hoc analysis of other headache disorders
Source: BMC Neurol. 2024 May 25;24:176. doi: 10.1186/s12883-024-03675-3 (PMC11127369; doi:10.1186/s12883-024-03675-3)
Supplement: Supplementary file 4 — Supplementary Material 4 [file 12883_2024_3675_MOESM4_ESM.docx]

## **Supplementary Data 4** Classification of cluster headache

| **Item^1^** | **Response** | **Patterns of responses** |
| --- | --- | --- |
| Headache lasting for 7 days to 1 year (single answer) | a) Yes  b) No | a) is selected |
| Duration (single answer) | a) <4 hours  b) Half a day  c) All day  d) 2 to 3 days  e) 4 to 14 days  f) ≥15 days | a) is selected |
| Site of pain (multiple answers) | a) Unilateral  b) Bilateral  c) Frontal  d) Occipital  e) Periorbital  f) Other | a) and c)  a) and e)  a), c), and e)  One of the above combinations is selected |
| State when in pain (single answer) | a) It is more comfortable to stay still  b) Staying still does not change the severity of pain  c) Pain makes it hard to stay still  d) I don't know | At least c) in “State when in pain” or at least e), f), or g) in “Symptom associated with headache” is selected |
| Symptom associated with headache (multiple answers) | a) Nausea or vomiting  b) Photophobia  c) Phonophobia  d) Osmophobia  e) Bloodshot eye on the side of headache  f) Teary eye on the side of headache  g) Runny nose on the side of headache  h) Dizziness  i) Weakness or lethargy  j) Stiff shoulders  k) Stiff neck  l) Numbness in hands and feet  m) Other |  |
| Severity (single answer) | a) No pain  b) Little pain  c) Moderate pain  d) Quite a bit of pain  e) Extreme pain | At least d) or e) is selected |

**Notes:** This study used the classification used in Sakai et al. [1].

^1^ If only one of the six criteria above did not apply, the patient was considered to have a "probable cluster headache" and was included in the cluster headache category.

**Reference**

1. Sakai F, Hirata K, Igarashi H, Takeshima T, Nakayama T, Sano H, et al. (submitted) A survey conducted using health claims records and questionnaires to investigate the prevalence of migraine in Japan. [Submitted to the Journal of Headache and Pain].
